# Visualized Automatic Feedback in Virtual Teams

**DOI:** 10.3389/fpsyg.2019.00814

**Published:** 2019-04-16

**Authors:** Ella Glikson, Anita W. Woolley, Pranav Gupta, Young Ji Kim

**Affiliations:** ^1^Tepper School of Business, Carnegie Mellon University, Pittsburgh, PA, United States; ^2^Department of Communication, University of California, Santa Barbara, Santa Barbara, CA, United States

**Keywords:** virtual team, task effort, feedback, team composition, conscientiousness, awareness systems

## Abstract

Management of effort is one of the biggest challenges in any team, and is particularly difficult in distributed teams, where behavior is relatively invisible to teammates. Awareness systems, which provide real-time visual feedback about team members’ behavior, may serve as an effective intervention tool for mitigating various sources of process-loss in teams, including team effort. However, most of the research on visualization tools has been focusing on team communication and learning, and their impact on team effort and consequently team performance has been hardly studied. Furthermore, this line of research has rarely addressed the way visualization tool may interact with team composition, while comprehension of this interaction may facilitate a conceptualization of more effective interventions. In this article we review the research on feedback in distributed teams and integrate it with the research on awareness systems. Focusing on team effort, we examine the effect of an effort visualization tool on team performance in 72 geographically distributed virtual project teams. In addition, we test the moderating effect of team composition, specifically team members’ conscientiousness, on the effectiveness of the effort visualization tool. Our findings demonstrate that the effort visualization tool increases team effort and improves the performance in teams with a low proportion of highly conscientious members, but not in teams with a high proportion of highly conscientious members. We discuss the theoretical and practical implications of our findings, and suggest the need of future research to address the way technological advances may contribute to management and research of team processes.

## Introduction

Measuring and managing the relative effort of contributors to a shared outcome is among the oldest problems in psychology (Triplett, 1898; Ringelmann, 1913). With the advent of technology and growth in technology-mediated collaboration in teams, the problem gets more complicated. Advances in information and communication technologies and continuing globalization keep facilitating the growing reliance of organizations on geographically dispersed virtual teams (Marlow et al., 2017). Geographical dispersion suggests dependence on technology for team communication and teamwork, which has dramatically changed team dynamics and processes (Breuer et al., 2016). Despite the significant use of geographically-dispersed virtual teams in organizations for tasks that require diverse expertise, knowledge and resources, the questions regarding how to enhance their performance are still open (Gilson et al., 2014).

One of the biggest challenges that dispersed virtual teams face is the management of team members’ effort (Peñarroja et al., 2017). The low visibility of team members’ individual contribution suggests a difficulty for social comparison and for monitoring and evaluation of each other’s effort. Under these circumstances team members might withhold their contribution to teamwork, resulting in significant process loss, or what is also known as social loafing (Ingham et al., 1974; Harkins and Szymanski, 1989).

Past research has demonstrated that feedback can be useful for increasing team motivation and reducing social loafing within distributed teams (Chidambaram and Tung, 2005). However, most of the current literature on feedback in teams suggests that it is very subjective and is typically given in a relatively complex one-time intervention that requires a focused session of team reflection to be effective (Konradt et al., 2015; Peñarroja et al., 2017). The relatively high cost (in terms of time and effort) and low effectiveness of existing feedback systems suggests a need for alternative ways of increasing team members’ motivation and effort.

Awareness tools may meet this need. For instance, the evolving literature on team awareness in collaborative learning suggests that dynamic tools that allow the members of dispersed teams to learn about the timing of each other’s activities and contributions may significantly improve team coordination and learning (Leinonen et al., 2005; Bodemer and Dehler, 2011; Buder, 2011). However, in these studies, awareness tools were mostly used for reflecting upon team members’ relative contribution to communication (DiMicco et al., 2007; Janssen et al., 2011), and have produced inconsistent findings (Jermann and Dillenbourg, 2008). Following this evolving line of research we suggest that using a shared, automatic, effort visualization tool that reflects member participation may regulate team members’ effort, thereby reducing social loafing and contributing to team performance.

Furthermore, we argue that such automatic feedback may not be useful for all teams, and that team composition will moderate its effectiveness. Specifically, we suggest that for teams with higher internal motivation (as a result of team members’ high conscientiousness), an effort visualization tool, aimed to increase external motivation, will be less effective than for teams with low internal motivation. To test our hypotheses regarding the effect of an effort visualization tool on team effort and performance, and the moderating role of team members’ internal motivation on the relations between the tool and team effort, we conducted an experiment. We examined the effect of the tool on the effort and performance of geographically distributed MBA students as they worked together on the Test of Collective Intelligence (TCI: Kim et al., 2017), a set of synchronous games designed to measure how well a group works together. As the teams completed the TCI we tested the moderating role of team members’ conscientiousness on the impact of the effort visualization tool on team effort and performance. In the next section we review past research on task effort, awareness tools, feedback, and team members’ internal motivation. By integrating these different streams of research and providing an empirical test of the proposed model, this article suggests a new approach for both researching and intervening in the distribution of effort in teams.

### Task Effort

Effort is a limited-capacity resource that could be allocated to a range of task-relevant and task-irrelevant activities (Yeo and Neal, 2004). Management research has long connected employees’ investment of intense task-relevant effort to successful job performance (Hackman, 1987; Blau, 1993; Byrne et al., 2005; Salas et al., 2005). In investigating the motivational factors leading to individuals’ tendency to invest or withhold task-relevant effort in teams, research has addressed both team composition, or the individual traits that enhance motivation and task-related effort (van Vianen and De Dreu, 2001; Judge and Ilies, 2002; LePine, 2003), as well as the characteristics of the social context (Latané, 1981; Kidwell and Bennett, 1993).

Chief among the team composition factors investigated with respect to effort is the individual characteristic of conscientiousness, shown to affect both motivation and task-oriented effort (Bell, 2007). Conscientiousness has been found to correlate with commitment, diligence, performance motivation and self-regulation in individual work and in collaboration (Humphrey et al., 2007; Kelsen and Liang, 2018). In terms of social context influence, despite the positive motivational aspect of conducting work in a group setting (Hart et al., 2004), research has demonstrated that the social context of teams, where others can do the work, tends to reduce individuals’ effort (Ingham et al., 1974). The tendency to make less effort when working in a team in comparison to working alone is known as social loafing (Latané et al., 1979).

Social loafing might vary across teams and is highly dependent on team characteristics such as team members’ geographic dispersion (Chidambaram and Tung, 2005; Blaskovich, 2008), which makes members more anonymous and their contributions less observable. The growing use of geographically dispersed virtual teams in contemporary organizations highlights the need to better understand the phenomenon of social loafing in this setting and effective ways to decrease it.

### Task Effort in Distributed Teams

Past research has identified several reasons behind the increased tendency of team members to withhold effort in distributed teams. For instance, Chidambaram and Tung (2005) suggested that the negative impact of team members’ dispersion on effort could be explained by the immediacy gap. Building upon Social Impact Theory (Latané, 1981) and research on social loafing (Kidwell and Bennett, 1993), Chidambaram and Tung argued that when members of a group become more isolated (and hence less immediate) their participation in and contribution to a group decreases. The immediacy gap relates to the difficulty in making social comparisons, which in turn decreases the salience of other members and their actions (Weisband, 2002). Comparing between collocated and dispersed virtual teams, Chidambaram and Tung (2005) found that physical dispersion, while not affecting the quality of the ideas teams produced, decreases the team members’ effort - the relative quantity of the produced ideas per team member, which in turn harms decision quality (performance).

Blaskovich (2008), also building upon Social Impact theory, suggested that the reliance on technology in distributed teams decreases the social impact and thus allows team members to disengage from the group, assuming the disengagement is not visible. Blaskovich (2008) found lower cognitive effort among members of the distributed teams in comparison to collocated teams. The time spent on the task did not differ, yet members in the dispersed teams were less attentive to the details of the task, and reported investing less effort.

Alnuaimi et al. (2010) followed Karau and Williams (1993) model of “collective effort,” as well as Bandura’s notion of moral disengagement (Bandura et al., 1996) and directly examined three possible mechanisms that might explain the impact of geographical dispersion on the tendency to withhold effort: attribution of blame, diffusion of responsibility and dehumanization. Their findings suggest that social loafing, driven by team members’ dispersion, was partially mediated by the dehumanization of the other team members, which was driven by the low identifiability of the distant teammates.

While team members’ relative anonymity may play an essential role in the low social presence of distant teammates and the consequent withholding of effort, the specific focus of the social comparisons and monitoring may also be essential (Salas et al., 2000). For instance, Mulvey and Klein (1998) argued that the actual withholding of effort may differ from the perceived team effort, with perceptions being the main driver of team motivation. Reasoning that team members might be particularly averse to carrying the workload while others free ride (Kerr, 1983), Mulvey and Klein (1998) found a significant negative relationship between perceived social loafing and team performance.

In a similar vein, Peñarroja et al. (2017) suggested that the inability of distributed team members to observe and monitor each other’s actual effort leads to greater reliance on assumptions and perceptions, which could be biased and erroneously negative. Researchers also noted that in order to correct the inaccuracy of the perceptions of social loafing thereby decreasing the overall withholding of effort and increasing team performance, teams need trustworthy feedback regarding its’ effort-related processes (Geister et al., 2006; Peñarroja et al., 2017).

### Team Feedback in Distributed Teams

Team feedback is defined as communication of information provided by (an) external agent(s) concerning actions, events, processes, or behaviors relative to task completion or teamwork (Gabelica et al., 2012). Performance feedback is conceptualized as the provision of information about individual or group outcomes, and process feedback is defined as information regarding the way one is performing a task, and thus relates to team dynamics, including team effort (Salas et al., 2012). Despite the overall value of feedback for increasing team effort and performance, its effectiveness is known to be limited (Kluger and DeNisi, 1996). Performance feedback in geographically dispersed teams has been explored with the intention to overcome the relative anonymity of individual effort driven by geographical dispersion. For instance, Fang and Chang (2014) looked at the effect of performance feedback, but found no significant difference between the outcomes of identifiable versus unidentifiable (anonymous) contributors. Similarly, Suleiman and Watson (2008) did not find an effect of identifiability or performance feedback on team members’ social loafing. Looking into the elements of social comparison, Chen et al. (2014) found that feedback regarding others’ high performance increased the effort of those whose contribution was identifiable, but not for unidentifiable participants.

As geographical distribution impacts the visibility of team members’ effort and motivation, Geister et al. (2006) suggested that process feedback could be especially useful for assessing others’ contribution, and thus minimizing social loafing. Peñarroja et al. (2017) provided feedback on both performance and process, and helped participants to understand the feedback via a session of guided reflexivity. They found that feedback decreased the perceptions of social loafing, which in turn increased team cohesion. Geister et al. (2006) tested the effect of an online process feedback system on team members’ motivation and performance. Their findings demonstrate that process feedback is useful for increasing trust and the effort of the least motivated team member (Geister et al., 2006).

A recent review of the impact of process and performance feedback recognized specific limitations to the efficiency of feedback, such as feedback timing, level of sharedness and feedback valence (Gabelica et al., 2012). Delayed feedback has less impact on team motivation than immediate feedback (e.g., Kerr et al., 2005). Feedback information only available to specific individual team members is less effective than feedback available to all team members (e.g., Barr and Conlon, 1994). And feedback communicated with a negative tone has been shown to have a negative effect on team processes (e.g., Peterson and Behfar, 2003).

While most of the studies on the effect of feedback were conducted in collocated teams, the technology that supports collaboration among geographically-dispersed team members may provide feedback that overcomes these limitations. Specifically, collaborative platforms may provide a vehicle for process feedback that (1) is automatically generated as team interaction is happening, and therefore is immediate; (2) is displayed on the shared platform, and thus is accessible to all team members; and (3) is visual, in that it does not rely on specific wording that often reflects a positive or negative tone. Existing research on this type of feedback to date has been conducted largely by researchers in education and technology, who examine the impact of awareness systems on team learning and communication in classroom settings. In the next section we provide a short review of this literature, and highlight the opportunity it provides for understanding the effect of feedback on effort and social loafing in geographically distributed teams.

### Team Awareness Systems and Visualization Tools

Team awareness refers to the ability to know what is going on in a team in real time. It helps the development of dynamic knowledge that is acquired and maintained via interactions within a team, and as a secondary goal, it aims to reflect process and assists in accomplishing a task (Gutwin and Greenberg, 2002, p. 416). Team awareness systems were developed to overcome the limitations of dispersed learning teams that use technology to communicate, thereby improving team processes and outcomes. Aiming to bring to awareness the hidden or unconscious team members’ behaviors, such as dominating a team conversation, team awareness systems are mostly used in the field of computer-supported collaborative work (CSCW, Gutwin and Greenberg, 2002) computer-supported collaborative learning (CSCL, Bodemer and Dehler, 2011) and group support systems (GSS, Dennis, 1996). However, the GSS research has rarely addressed the impact of team awareness on team effort or performance (Briggs et al., 2003).

Many awareness systems use visualization tools, as visualization produces an easier way to display and interpret complex and extensive information than verbal description (Ware, 2005). Specifically, visualization is typically used to reflect relative team member participation in communication-related activities (Janssen et al., 2007, 2011; Jermann and Dillenbourg, 2008; Kim et al., 2012). For instance, DiMicco et al. (2004) showed participants a graph that reflected the relative participation of each team member in a discussion. Jermann and Dillenbourg (2008) compared the effect of tools which reflected the relative or cumulated team members’ contribution to a specific discussion topic. Streng et al. (2009) created a visualization of the quality of a discussion, measuring it in comparison to a pre-scripted discussion structure. They compared a diagram-like visualization that included graphs and figures, with a metaphoric picture, in which objects represented the discussants’ roles (Streng et al., 2009). Similarly, Leshed et al. (2010), also aiming to reflect discussion quality, visualized the relative use of specific words categorized to themes, such as emotional or self-reference words, and compared the effect of visualization by bar-charts with visualization via an animated image.

Despite the common notion that visualizations mirror team participation across these studies, the empirical findings vary with respect to their effect on regulating effort and performance. For example, aiming to reach more equality in discussion, and examining the discourse of collocated teams, DiMicco et al. (2004) found that presenting the relative team members’ contribution to a discussion significantly reduced the amount of speech of the most active team member, but had no effect on the least active team member. Kim et al. (2012) examined collocated and distributed teams, and found that a representation of team members’ relative contribution to a conversation increased the overall discussion volume, and improved the level of cooperation among distributed team members. Similarly, Janssen et al. (2011) examined the effect of time that team members spent looking at the participation visualization, and found that time spent with the tool increased the amount of participation in online discussion, as well as the equality of participation among the team members, however, no effect was found on the actual team performance. Streng et al. (2009) found that metaphoric representation was more effective than chart-like representation and led to a quicker change in undesirable behavior. In contrast, Jermann and Dillenbourg (2008) did not find any effect of a visualization tool.

These inconsistencies draw attention to several distinctions among the mentioned visualization tools. The first distinction relates to the subjective versus objective reflection of teamwork. While some studies presented participants with the reflection of their actual measured level of participation (e.g., Janssen et al., 2007), others presented team members with subjective perceptions of participation (Geister et al., 2006). The subjective perception (peer feedback), though highly important, does not allow for a continuous immediate reflection of one’s own action, and as a result of subjectivity could be viewed as biased and distrusted by team members. The second distinction refers to how behavior was represented; some of the tools emphasized the amount of actual behavior (Janssen et al., 2007), thus increasing awareness of team processes, while others were more focused on the gap between the actual and the desirable behaviors for the task at hand (e.g., Streng et al., 2009). The establishment of a normative standard to which to compare team behavior interjects the same drawbacks that exist for more traditional verbal feedback: elements of subjectivity and context specificity. In contrast, automatic visualization of self and others’ effort should provide a more objective, valence-free feedback that increases team awareness, with less backlash due to a sense of subjectivity or manipulation. Over-complexity or over-gamification of the representation could also be a drawback, as it may draw more attention toward understanding the tool than to the actual teamwork (Leshed et al., 2010).

Balanced discussion that aims at equally distributed communication means reducing the contribution of the over-participator (DiMicco et al., 2004). In contrast, balancing team members’ effort on the work itself aims at reducing social loafing, which potentially means increasing the contribution of all team members, and especially the least contributing member. Thus, we suggest that automatic and dynamic visualization of team members’ actual task-related effort will increase team members’ awareness of other members’ effort, and serve as an external motivator to increase the overall level of team task-related effort.

H1: A visualization of the relative team members’ effort will increase overall team effort.

### The Moderating Role of Team Composition

Examining feedback on the individual and team levels, researchers have long conceptualized that the effectiveness of feedback depends on team composition (e.g., DeShon et al., 2004). Team members’ abilities and predispositions influence both the actual team processes, as well as the ability to adjust to feedback. These differences could partially explain the inconsistency of feedback effectiveness documented in previous studies (Kluger and DeNisi, 1996). Nevertheless, existing team research has rarely studied the interaction of feedback and team composition.

Technological development facilitates the evolution of support systems, which are capable of visualizing team members’ effort. These capabilities provide a relatively easy and inexpensive way to increase team awareness in distributed teams. In addition, this mode of feedback can be easily altered and managed, such as by switching it on or off, or moving from team to individual level and vice versa. Thus, the use of such a tool could be adjusted to a specific team, taking into consideration team members’ predisposition and their initial motivation.

Building on the demonstrated importance of intrinsic motivation for reducing social loafing and increasing team effort (George, 1992), team composition researchers have looked at team members’ personality trait of conscientiousness (Bell, 2007; Hoon and Tan, 2008). Conscientiousness refers to the extent to which a person is self-disciplined and organized (Costa and McCrae, 1992), and has been found as the most consistent predictor of individual performance (Hurtz and Donovan, 2000; Salgado, 2003). Peeters et al. (2006) meta-analysis supported the claim that team members’ conscientiousness is positively related to team performance in professional and student teams. Looking to explain the mechanism through which conscientiousness influences team performance, researchers found that it is negatively related to social loafing (Ferrari and Pychyl Timothy, 2012; Schippers, 2014). Furthermore, Schippers (2014) found that teams with high levels of conscientiousness were able to overcome the negative effects of social loafing, as highly conscientious members compensated for the lack of effort of other teammates. This means that teams with a high proportion of conscientious members may demonstrate high levels of motivation and effort regardless of the visibility of their and other members’ effort. George (1992) demonstrated that when intrinsic motivation was low, task visibility significantly lowered social loafing. However, when intrinsic motivation was high, task visibility had no effect on team effort. Bringing these lines of research together, we suggest that effort visualization tools represent a way to enhance team extrinsic motivation via social comparison, and team members’ conscientiousness represents team members’ internal motivation. Thus, teams with a majority of members low in conscientiousness will have lower internal motivation and are likely to benefit more from an extrinsic motivation-inducing visualization of team effort, than teams where most members are high in conscientiousness. Raising awareness of the effort of other members can augment the motivation of members who are low in conscientiousness and reduce the withholding of task-oriented effort.

H2: The impact of an effort visualization tool on team effort will be moderated by team composition, such that the effort visualization tool will increase team effort in teams with a low number of highly conscientious members, but not in teams where most members are highly conscientious.

Building on the literature that connects effort to team performance (Hackman, 1987; Yeo and Neal, 2004; Byrne et al., 2005), we suggest that by increasing team members’ effort, a visualization tool focused on team effort will contribute to team performance. However, this effect will be moderated by team composition. Thus, we predict the following:

H3: The impact of an effort visualization tool on performance will be mediated by team effort and moderated by team composition.

## Materials and Methods

### Sample and Procedure

We randomly assigned 335 MBA students to 80 distributed virtual project teams (3–4 members) as part of a cross-cultural management course. Males comprised 55% of the sample, and the average age was 29.23 years old (*SD* = 8.23). All teams had members located across different countries (geographically dispersed), with no previous familiarity. At the beginning of the project, participants individually completed a survey assessing their demographics and personality traits. As part of the team project, members of each team worked together to complete the Test of Collective Intelligence (TCI; Kim et al., 2017), which includes eight collaborative tasks. All teams were randomly assigned to one of the two conditions: effort visualization tool condition or control condition. Due to different technical problems experienced by eight teams, the final number of teams included in the study is 72. During the team task (TCI), team members’ effort was objectively measured. Team performance was measured as the aggregate t performance on all of the TCI tasks^1^. The data was collected under approval of Behavioral Sciences Research Ethics Committee, Technion – Israel Institute of Technology.

### Manipulations and Measures

**FIGURE 1:**
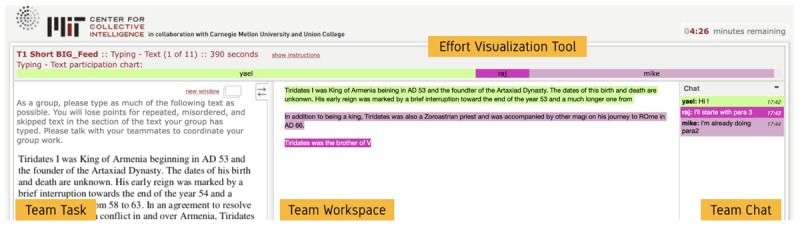
Illustration of the effort visualization tool. The bars represent each team member by color and the name. Bars automatically change their width based on the relative effort operationalized as the real-time count of valid keystrokes.

*Effort visualization tool*: Building upon the Platform for Online Group Studies (POGS; Kim et al., 2017) we integrated a visual awareness system, which reflected the relative effort of team members based on the number of keystrokes they made within the task collaboration space. Whenever a team member would type within the workspace the proportion of their contribution to the team’s work product was calculated relative to other team members and displayed as a bar across the top of the screen. Each team member is indicated by their unique color, which was also used to highlight the members’ keystrokes in the workspace. The more a team member contributed relative to other team members, the wider their colored bar got in real time (see Figure 1).

*Team effort* was operationalized by aggregating the total number of keystrokes made by the members of a given team while interacting with the tasks comprised in the TCI. The average number of keystrokes was 1468.35 per team (*SD* = 408.86). For correlations of the measure with performance and other variables see Table 1. The average number of keystrokes made by the most contributing team member *M_(max)_* = 566.10 (*SD* = 116.19) which was significantly higher than the average of the keystrokes made by the least contributing team member *M_(min)_* = 227.06 (*SD* = 156.13) *t*(71) = 16.15, *p* < 0.05.

**Table 1:** Means, standard deviations and correlations (team-level variables).

	Mean	*SD*	1	2	3	4	5	6
(1) Performance	0.01	0.51						
(2) Effort visualization tool^1^	0.49	0.50	0.09					
(3) Team effort	1468.35	408.86	0.46**	0.29*				
(4) Team composition^2^	0.36	0.28	-0.04	-0.13	-0.06			
(5) Team size	3.88	0.33	0.29*	0.03	0.14	0.04		
(6) Proportion of females	0.36	0.28	-0.25*	-0.07	0.05	0.19	-0.19	
(7) English proficiency	6.17	0.40	0.26*	-0.01	0.18	-0.13	-0.03	0.04

*^∗^*p* < 05; ^∗∗^*p* < 0.01. *N* = 72. Team performance was standardized according to the TCI procedure (Kim et al., 2017).*

*^*1*^Effort visualization tool is a binary indicator of our experimental manipulation (0 = not present, 1 = present) and therefore correlations with this variable are Point-Biserial correlations; all remaining correlations are Pearson Bivariate.*

*^*2*^Team composition is indexed here as proportion of highly conscientious members.*

*Performance* was measured as the team’s score on TCI. The TCI includes eight collaborative tasks, designed to capture diverse group processes (e.g., generating, memorizing, problem solving, and executing; Engel et al., 2014; Kim et al., 2017). For example, for the generating task, team members had to brainstorm as many ideas as they could for the usage of a brick. The memorizing task required team members to remember words placed in grids of various sizes and reproduce the word grids together. An example of problem solving tasks includes solving matrix reasoning puzzles similar to Raven’s Progressive Matrices. To measure teams’ executing process, we used a typing task where teams had to copy as much and as accurately as possible from paragraphs of text. The TCI score is a weighted average of the teams’ task scores with the weights chosen to maximize correlation with all the tasks. The measured reliability of the TCI was Cronbach’s alpha = 0.68. An advantage of using the TCI to measure team performance is that it focuses on a holistic measure of groups’ ability to work together across different types of tasks (teams’ collective intelligence), which more reliably generalizes to and predicts teams’ future performance than performance on a single task (Kim et al., 2017).

*Team composition* was measured by calculating the proportion of highly conscientious team members. Conscientiousness was measured on the individual level using the FFM scale (Gosling et al., 2003). The measured reliability of the scale was Cronbach’s alpha = 0.71. The sample of participants was (median) split into two categories: highly and low conscientiousness (*Median* = 4, on 5 items Likert-like scale; 1 - not at all, 5 - to a great extent; *M* = 3.95, *SD* = 0.83). After categorizing individual participants, the proportion of highly conscientiousness members was calculated for each team. This has been shown to be a better representation of the presence of a trait in a team compared to looking at team mean levels as it factors in the number of different people who possess the trait at a high or low level (for similar procedure, see Miron-Spektor et al., 2011).

*Control variables* used in analyses included the number of team members (3 or 4), proportion of females in the team and team members’ level of English proficiency (measured by self-evaluation, 1 = not proficient; 7 = fluent, overall average = 6.08).

## Results

Descriptive statistics and correlations among variables are presented in Table 1.

The first hypothesis regarding the effect of a visualization tool on team members’ effort was tested using a hierarchical regression model and revealed a significant effect of the visualization tool on team effort (*b* = 239.33, *SE* = 92.48, *p* = 0.01; see Table 2; Model 3). The effect of team composition on team effort was insignificant (Table 2; Model 3). The moderating effect of team composition (H2) was significant (*b* = -826.33, *SE* = 335.99, *p* = 0.02, see Table 2; Model 4).

**Table 2:** Hierarchical regression model for team effort.

	Model 1 (Controls)	Model 2	Model 3	Model 4
Team size	0.163	0.157	0.163	0.151
Proportion of female team members	0.074	0.094	0.107	0.119
English proficiency	0.178	0.179	0.183	0.146
Effort visualization tool		0.295*	0.287*	0.647*
Team composition			-0.064	0.158
Visualization × Composition				-0.48*
*F*	1.41	2.84*	2.31	3.08*
*p-value*	0.25	0.03	0.05	0.01
*R^2^*	0.06	0.15	0.15	0.22
*Adj R^2^*	0.02	0.09	0.08	0.15
*Significance of F change*	0.25	0.01	0.59	0.02

*^∗^*p* < 0.05*

**FIGURE 2:**
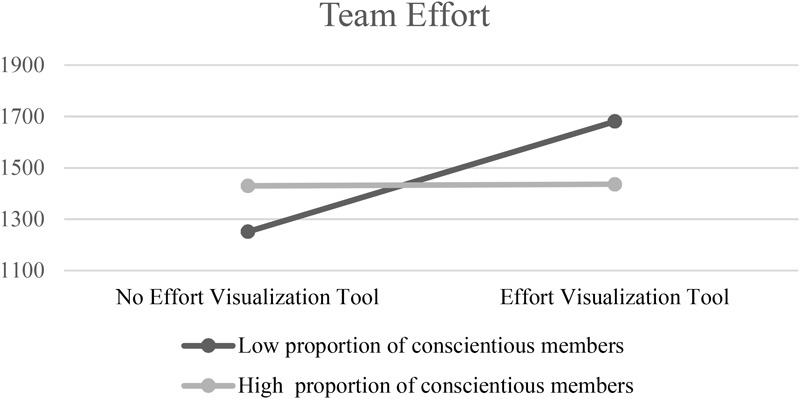
The moderating effect of team composition on the impact of effort visualization tool on team members’ effort.

Looking into the team composition distribution, we found that almost half of the teams (38 out or 72) had none or only one highly conscientious team member. Splitting the sample based on this characteristic allowed us to gain a better understanding of the interaction. Following Aiken and West (1991) we conducted simple slopes analysis, which revealed that for teams with a low percentage of highly conscientious members (i.e., teams with 0 or one highly conscientious team member) the impact of effort visualization tool led to a significant increase in team effort (*b* = 291.94, *SE* = 131.10, *p* < 0.05). However, for teams with a higher percentage of highly conscientious team members the impact of effort visualization tool was not significant (*b* = - 28.67, *SE* = 140.03, *p* = 0.84; see Figure 2).

Although we did not articulate a specific hypothesis regarding the effect of the effort visualization tool on highest and lowest team contributor, we suspected that due to the visualization of the relative effort social comparisons would become easier and therefore the effort visualization would increase the effort of the lowest contributor, but not the effort of the highest contributor. Indeed the results indicate that for the highest contributor there was no significant direct effect of the effort visualization tool, and no significant moderation effect of team composition. In contrast, for the lowest contributor the direct effect of the effort visualization tool was significant [*F*(1,70) = 3.69, *p* < 0.07]; lowest contributor without the tool (*M_(min)_* = 193.32; *SD* = 149.28; lowest contributor with the tool (*M_(min)_* = 262.71; *SD* = 157.36)). The moderation effect of team composition on the effort of the lowest contributor was also significant [*F*(3,68) = 4.00, *p* < 0.05] and similar to what we found for the total amount of contribution. We observed a significant effect of the effort visualization tool on the effort of the lowest contributor for teams with a low proportion of highly conscientious members (simple slope for -1SD; *b* = 110.42, *SE* = 51.47, *p* < 0.05), and an insignificant effect of the effort visualization tool on the effort of the lowest contributor for teams with a high proportion of highly conscientious members (simple slope +1SD; *b* = -14.96, *SE* = 51.62, *p* = 0.77).

To further validate our findings we looked at the variance in effort within teams, measured as standard deviation of the effort. The effort visualization tool and proportion of highly conscientious members each had no direct effect on the variance in effort within teams, however the interaction of them was significant [*F*(3,68) = 2.69, *p* < 0.07], and revealed that effort visualization tool had a significant negative effect on the variance in effort for teams with a low proportion of highly conscientious members (simple slope for -1SD; *b* = -0.04, *SE* = 0.02, *p* < 0.07) such that the effort visualization reduces the variance in effort in teams with fewer highly conscientious members. For teams with a high proportion of highly conscientious members the effect of the effort visualization tool was insignificant (simple slope for +1SD; *b* = 0.02, *SE* = 0.02; *p* = 0.24).

The third hypothesis suggested that the impact of the visualization tool on performance will be mediated by team effort and moderated by team composition. First, we examined the effect of the effort visualization tool and team conscientiousness on team performance. The results demonstrated a similar effect as found for team effort: the interaction effect was significant [*b* = 0.44; *F*(5,66) = 2.99, *p* < 0.05]. Similar to the effect on effort, the effort visualization led to a significant increase in team performance for teams with a low percentage of highly conscientious members (*b* = 0.35, *SE* = 0.17, *p* < 0.05), but not for teams with high percentage of highly conscientious members (*b* = -0.20, *SE* = 0.18, *p* = 0.14). The moderated mediation model was tested using bootstrap sampling produced by PROCESS macro in SPSS (Model 7; Hayes, 2013) and was significant [*F*(6,65) = 2.72, *p* < 0.05, *R*^2^ = 0.20]. Specifically the mediation was significant for teams with a low proportion of highly conscientious team members [CI 95% *b* = 545.64, *SE* = 150.71, *p* < 0.001, *LL-UL* (244.64; 846.64)], but not for teams with a high proportion of highly conscientious members [CI 95% *b* = -116.75, *SE* = 162.10, *p* = 0.47, *LL-UL* (-44.48; 206.88)].

## Discussion

The management of task-oriented effort in teams provides a great challenge for managers and researchers. While process feedback remains the most effective intervention for inducing task-oriented effort (Peñarroja et al., 2017), its availability and delivery could dramatically change, based on current technological developments (Streng et al., 2009; Leshed et al., 2010). Integrating the knowledge of the importance of one’s perceptions for regulating self-effort (e.g., Mulvey and Klein, 1998), with the literature on computer-mediated collaboration awareness systems (e.g., Bodemer and Dehler, 2011), this study demonstrated a way the visualization of team member effort may serve to provide efficient and effective process feedback.

In addition, incorporating team composition research that suggests the impact of team members’ traits on team motivation and effort (e.g., Bell, 2007), we theorized and found a moderating role of team members’ conscientiousness on the effect of the visualization tool on team effort and performance. Specifically, we found that the visualization tool was effective for teams with a low proportion of highly conscientious members, but not for teams with a high proportion of highly conscientious members. Thus, we have also demonstrated a boundary condition for this type of process feedback, based on team composition characteristics. We suggest that our study serves as an example for effective visualized process feedback, which when targeted appropriately based on team composition, may facilitate the effort and performance in geographically distributed virtual teams.

This study makes several theoretical contributions. First, it bridges several research streams which address task effort from different perspectives. By integrating the literature on social loafing (Chidambaram and Tung, 2005), team perceptions (Peñarroja et al., 2017), and feedback and awareness systems (e.g., Janssen et al., 2011), we demonstrated the positive role that automatic visualization may play in facilitating task effort. Research on social loafing addresses the role of social comparison, identification and fairness in understanding one’s effort in the context of teamwork (Mulvey and Klein, 1998; Alnuaimi et al., 2010). Illuminating these subjective processes, this line of research suggests a need for external intervention, which may influence or correct these perceptions via feedback (Peñarroja et al., 2017; Salas et al., 2008). However, the external facilitation required to produce effective integration of traditional feedback might limit its use due to the associated effort and cost required. At the same time, technological developments give us the ability to produce automatic visualized feedback (DiMicco et al., 2004; Jermann and Dillenbourg, 2008). This type of feedback has been studied mostly by education and technology researchers, and has not yet gained popularity among teams’ researchers. Integrating these new developments within the existing streams of research opens an opportunity for future research that may suggest different conceptualizations and operationalizations of process feedback, reflecting both the available technology and the aggregated past knowledge.

In addition, this study draws on the team composition literature (Peeters et al., 2006; Bell, 2007; Kelsen and Liang, 2018), and illustrates the need to address team composition when considering feedback interventions, by examining the moderating effect of team members’ conscientiousness on the effectiveness of the visualization tool. While technological developments open up the possibility of providing feedback in automated ways, such an approach requires strong and empirically-supported theories demonstrating the fit of feedback tools to a given team composition. Integrating these lines of research would allow for a better understanding of the interaction between internal and external motivations within a team, and their implications for team processes and performance.

Finally, this study emphasizes the importance of team processes and the potential for process feedback. The developing technology enables researchers and leaders to capture different aspects of team process, such as team effort, which were previously largely tacit and unobservable or solely reliant on team self-report. Embracing these abilities may contribute to a more profound understanding of team process and its responsiveness to process feedback.

### Practical Implications

This study suggests two main practical implications. First, it presents how visualization of team members’ effort may reduce social loafing in distributed virtual teams. Using an automatic visualization may encourage team members to put more effort into their work, decreasing the misperceptions regarding other members’ under-participation. The use of such a tool could be especially effective for encouraging the effort of the least contributing member of the team (Geister et al., 2006).

In more general terms, technology provides new ways for capturing, measuring and managing team members’ effort. While in the past, effort was an elusive factor that was highly difficult to measure, today any computerized work allows for the possibility that effort could be objectively assessed and managed (e.g., Google Docs’ edit history, Slack’s workspace data). Nevertheless, it is important to note that technology use in teams can also activate negative mechanisms, producing adversarial and unintended consequences (Marjchrzak et al., 2013; Ter Hoeven et al., 2016). Effort does not always lead to better performance, and an abuse of “effort management” using technology may lead to loss of motivation, reactance, and unproductive behaviors. Therefore, there is a need for future research to suggest and test the effectiveness as well as the limitations of using technology to manage effort.

Our second practical implication relates to the need to fit process feedback to a team’s composition. As team facilitation in general and feedback in particular become more automated, there are more opportunities to address the specific needs of a team, based on its members’ characteristics. Our study demonstrates that the same effort visualization tool that is effective for teams with a low proportion of highly conscientious members is totally ineffective for teams with a high proportion of highly conscientious members. It is also possible that under some conditions, the same feedback will have the opposite effect. Looking into the future of team management, there is a growing need to understand what type of feedback would be more effective for different types of teams.

### Limitations and Future Research

This study provides an integration of different lines of research and an empirical study that demonstrated the effect of an effort visualization tool on team effort and performance. On a general note, visualization tools aimed to raise team members’ awareness may increase the overall sense of being observed, and thus might lead to increased effort simply due to the mere presence of an observer, or, on the flip side may evoke participant reactivity. Here, in the context of our laboratory study, all participants were being “observed,” only the additional information about relative effort was manipulated, and the response to that observation was indeed the effect of interest. Conversely, participant reactivity may lead to mixed results, including negative feelings, and to an intentional withholding of effort. In this study, we did not observe such reactions, as could be evident from the additional analyses described which demonstrated an overall increase in effort related to the effort visualization tool, along with a decrease in variance in team members’ effort. However, future studies need to address this possibility, and examine the factors which may evoke such reaction.

An additional limitation of this study relates to the fact that the visualization tool was used during a short-term intervention. Future research should examine the long-term effects of an effort visualization tool, to realize its potential for learning, as well as the potential habituation that could occur if it was present in an ongoing way.

In addition, automation provides a range of different types of visualization and presentation (Janssen et al., 2007; Jermann and Dillenbourg, 2008; Streng et al., 2009). In this study we tested only one way of visualizing the relative effort in teams. The evolving research on awareness systems had started to address the different aspects of visualization, such as use of metaphoric representation or animated images (e.g., Leshed et al., 2010). However, more interdisciplinary research is needed to address both the psychological and perception-related aspects of team reflection.

While we focused on team members’ conscientiousness due to its relation to team members’ internal motivation, other aspects of team composition may also play an important role for team members’ acceptance of visualized team effort. For instance, team members with more independent self-construal (Triandis, 1989) might be less responsive to the relative representation of team effort than team members with more interdependent self-construal. Furthermore, the timing of the intervention could also serve as a moderating factor. In some teams it could be useful to reflect the effort at the initial stage of teamwork, while in others, it could be more efficient to introduce such feedback after the initial relationships in team have been established.

## Conclusion

The purpose of this study was to present a developing area for the management of team effort via team visualization tools, thereby integrating new and more established lines of research from different disciplines, and to empirically test the effect of one such tool on effort and performance in geographically distributed teams. Consistent with our hypotheses, we found that the effect of team effort visualization tool was moderated by team composition, demonstrating that only teams with a low proportion of highly conscientious members benefited from the visualization. Integrating different lines of research, we demonstrate the way new technology enables objective, immediate, and visual process feedback, which may improve effort in geographically-distributed teams, and the way team composition moderates the effect of such feedback on team effort and consequently on team performance.

## Ethics Statement

The data was collected under approval of Behavioral Sciences Research Ethics Committee, “Technion – Israel Institute of Technology.”

## Author Contributions

EG, AW, PG, and YK contributed conception and design of the study. YK and PG made part of the statistical analyses. EG and AW wrote the first draft of the manuscript.

## Conflict of Interest Statement

The authors declare that the research was conducted in the absence of any commercial or financial relationships that could be construed as a potential conflict of interest.

## Footnotes

1 The data underlying the study is available per request.
